# The educational effects of emergency remote teaching practices—The case of covid-19 school closure in Italy

**DOI:** 10.1371/journal.pone.0280494

**Published:** 2023-01-25

**Authors:** Alice Bertoletti, Mara Soncin, Marta Cannistrà, Tommaso Agasisti

**Affiliations:** 1 School of Management, Politecnico di Milano, Milan, Italy; 2 European Commission, Joint Research Center, Seville, Spain; Central China Normal University, CHINA

## Abstract

The disruption caused by the Covid-19 pandemic generated an unprecedented situation, in which digital learning, in the form of Emergency Remote Teaching, was the only possible form of schooling. Italy soon decided to close schools as a measure to counter the spread of the virus. Although the Ministry of Education suggested general guidelines, school principals and teachers were autonomous in deciding how to best organise their educational activities. The first objective of our study is to identify subgroups of teachers, based on the use of digital tools during the Covid-19 emergency. Secondly, we explore how subgroups differ in terms of teachers’ satisfaction and students’ performance. To this end, we integrate information from an ad hoc survey completed by 1,407 primary and lower secondary teachers in Italy, with the students’ standardised test scores provided by INVALSI. Data have been analysed through a 3-step latent class analysis. The findings reveal that one third of the teachers showed resistance to the use of digital technologies and focused mainly on asynchronous teaching. Teachers that used a broader set of digital instruments, instead, were more satisfied with their teaching practices. A more articulated use of technology for teaching activities was also positively associated with higher students’ performance in 2021.

## Introduction

The disruption caused by the Covid-19 pandemic generated an unprecedented situation, in which emergency remote education was adopted globally to ensure educational continuity for 1.5 billion students and 63 million teachers [1]. In April 2020, more than 90% of worldwide educational systems were locked down. While most Western countries owned the technological infrastructure needed to deliver distance education successfully, heterogeneity in the level of teachers’ digital competence and readiness was much broader [2]. The teaching approach used during the Covid-19 emergency is defined as Emergency Remote Teaching (ERT) [3], with features in common with distance and digital learning but characterised by a completely different context of application (i.e., teaching and learning during a pandemic). A previous example of ERT comes from the SARS crisis in Asia in 2001, which, on the one hand, highlighted the potentialities of digital tools for learning but, on the other hand, showed the lack of preparation in facing such a complex situation [4].

Recent studies focused their attention on students’ learning conditions during the Covid-19 outbreak, trying to highlight the difficulties and disadvantages of distance learning in lockdown [2, 5, 6]. Exploiting surveys of teachers to collect data on perceptions and teaching practices, recent evidence stresses the central role of the teacher as the main mediator of the learning process, especially in times of emergency, with a re-valorisation of the teaching role [7, 8]. However, existing research did not exploit the heterogeneity in the use of technologies for ERT, despite the impact this may have on teaching quality [9]. Besides, existing literature explored the impact of Covid-19 disruption on teaching practices, on the one hand, and on student performance, on the other [10]. The link between the two aspects is worth of investigation, to understand the role of ERT in mitigating the potential drawbacks of school closure—which is still unclear [11]. The current research contributes to this strand by investigating teachers’ use of digital tools and its effects on two relevant outcomes: teachers’ satisfaction and students’ performance.

The context of application is the Italian scenario where the shutdown was enforced in late February 2020 and lasted until September, making Italy one of the countries affected by the longest school closure in the world [1]. Although the Ministry of Education suggested some general guidelines, school principals and teachers were autonomous in deciding how to best organise their educational activities. The resulting heterogeneity in practices and experiences is the focus of our study, whose specific research objectives are to (i) identify different subgroups of teachers on the basis of the use of digital tools during the COVID-19 emergency; (ii) investigate how these subgroups differ in terms of teachers’ satisfaction and students’ performance.

To pursue our objectives, a 3-step Latent Class Analysis (LCA) is employed to identify latent groups of teachers with similar digital behaviours during Covid-19 school closure. Besides, latent classes are characterised by means of teachers’ and contextual characteristics. Finally, we test the difference of two outcomes (i.e. teachers’ satisfaction and students’ performance) across latent classes. Data are collected through an *ad hoc* survey designed in partnership with the National Evaluation Committee for Education (INVALSI) and handed out to a nationally representative sample of primary and middle schools. The survey was carried out between July and September 2020, covering information about the use of ICT during the first wave of school closures (March-June 2020). Besides the survey data, we use information on INVALSI standardised test scores in 2021 to investigate the effect of digital tools used by teachers on students’ performance. To the best of our knowledge, this is the first academic paper that combines these two levels of investigation.

## Relevant literature

### Teachers’ satisfaction and digital tools

In the past two decades, several scholars addressed the use of ICT for teaching, underlining facilitators and barriers of the use of digital technologies [12–15]. Institutional support and ICT positive emotions are found to be positive mediators to ICT self-efficacy and work engagement [16]. Quality of teaching has been found to be positively related to a confident and critical use of ICT for teaching, especially when this is preceded by training [17]. If, in the pre-Covid era, the relationship between self-efficacy perception in the use of digital technologies and teachers’ satisfaction was positive [16, 18], the topic has been under-investigated during the emergency period.

Focusing specifically on teachers’ satisfaction during the Covid-19 pandemic, the literature provides interesting results. In general, the teachers’ well-being was negatively affected by the school closure imposed by the pandemic. During those months, teachers had to face disruptive and structural changes on short notice. Analysing the satisfaction of Portuguese teachers during the Covid-19 pandemic, [19] found that male teachers were the most penalised category, with long working hours and difficulties using digital technologies. On the other hand, the emergency situation reinforced collaborative practices among teachers through the creation of a network of colleagues supporting each other. These new communities enabled the creation of a sense of cohesion that helped to overcome the loneliness and psychological challenges that teachers had to face [6, 20, 21]. A report of the Joint Research Centre [6] analysed the schooling practices during the Covid-19 pandemic in five EU countries (i.e., Belgium, Estonia, Greece, Italy and Poland), highlighting the need to increase the digital competences. The quality of digital infrastructure and tools also emerges as a fundamental requirement for enabling high-quality education during an emergency (and beyond).

We contribute to this strand of research by exploring the relationship between the teachers’ use of digital tools and their satisfaction with teaching activities during the emergency. In this context, we use teachers’ satisfaction as a proxy, albeit subjective, for the quality of teaching carried out in ERT [18]. This issue is particularly relevant given the strong link between teachers’ well-being and the educational achievement of students (see, for instance, [22]).

### Students’ performance and digital tools

Research on how digital tools influence students’ outcomes is part of a relevant and growing stream of academic literature. The possibilities offered by technology in the educational setting are multiple: game-based activities [23], virtual laboratories [24] or computed-based scaffolding [25] represent only a small fraction of possible applications. Due to the diversity in the available digital tools, evidence on the effectiveness in terms of students’ outcomes is mixed, usually showing positive but modest effects [26]. Effect size increases if we consider applications in STEM disciplines [25–27]. Also, results are mixed if comparing different grade levels: the meta-analysis by [28] reported different effect sizes among grade levels, whereas other meta-analyses (e.g., [29]) found no differences in the effect of digital tools on student learning between different grade levels. Less evidence is available for primary schools, where the adoption of digital tools is more limited compared with higher grades. As an exception, the evaluation designed by [30] examined the effects of a digital formative assessment tool on mathematics achievement in grade 3. Results showed positive effects, especially for high-performing students.

The impact of educational technology in the classroom is highly debated among researchers and practitioners. Particularly, the link between the use of educational technology and students’ achievement is still unclear [31]. However, Covid-19 forced (almost all) schools to intensively adopt technology for educational purposes, despite evidence about its impact on students’ learning is still limited. In the EU, only a few studies assessed the effect of Covid-19 related school closure on primary and secondary students’ standardised test results [11]. [32] evaluated school closures on primary school performance using data from the national Dutch test. Findings reveal a learning loss of about 3 percentile points or 0.08 standard deviations. [10] evaluated the effects of school closures based on standardised tests in the last year of primary school in Flemish schools in Belgium. They found that students of the 2020 cohort experienced significant learning loss in all tested subjects, with a decrease in school averages of mathematics scores of 0.19 standard deviations and Dutch scores of 0.29 standard deviations as compared to the previous cohort. A related study [33] argued that low-achieving students may be particularly affected by the lack of educator’s support during school closures. Authors used instructional time as a proxy for students’ achievement, this being one of the most relevant factors affecting learning outcomes [34]. They found that while students, on average, reduced their daily learning time, the reduction was significantly larger for low-achievers than for high-achievers. Despite studies focused on estimating the impact of school closure on students’ performance, there is still a lack of knowledge on the role of digital tools in mitigating the potential learning loss of students [11].

This paper aims at filling this gap in the literature by linking teachers’ practices during the first months of Covid-19 emergency and students’ performance in standardised test scores collected in May 2021, focusing on the Italian educational system. Despite Italy was one of the European countries mostly affected by the Covid-19 emergency [1], quantitative evidence on the effect of school closure is still scarcely provided for the Italian context. Qualitative studies have shown that the reaction to school closure in Italy has been heterogeneous across institutions. Italian schools with previous experience in digital learning proved to be more ready to respond to the emergency [2]. In general, the most relevant criticalities were the existence of digital barriers among teachers and students, and an inefficient organisation of the school system [6, 35]. The replication of traditional teaching practices in an online learning environment revealed significant criticalities due to the unsuitability of these tools for the new schooling modalities [6]. The process of readjusting traditional learning strategies to online schooling caused an increase in the workload of teachers [2, 35]. Finally, as for other countries, remote learning in Italy was not able to reach every student equally. In particular, the most negatively affected students are potentially those with special education needs and the ones with low socioeconomic status, who are more likely to lack digital tools and strong family support [5, 6].

## Methodological approach

### Framework for modelling digital practices

The empirical analyses rely on a framework that models the digital practices implemented by teachers during ERT. In the literature, we observe two dimensions for classifying digital technologies for teaching [36, 37]: (1) the activities they support; (2) the level of simultaneity they involve. The first dimension entails the distinction between technologies used to support the learning activity, and the ones employed to communicate with students and families. The first category requires the creation of a proper online learning environment, which was rarely implemented in schools before the Covid-19 disruption [6]. While digital tools are more likely to have been used by teachers for the first time so intensively during the Covid-19 emergency, communication technologies were implemented even before the ERT period. However, the emergency made it necessary to increase the intensity of communication with students and families. Indeed, full-time remote schooling required a more active role of parents in mediating between teachers and students, especially at lower grades [6]. Constant communication between teachers, parents and students represented, therefore, an essential requirement for implementing remote teaching activities [38, 39]. The communication channels adopted by teachers to create a bridge between school and families were both conventional, such as email, and unconventional, such as private calling [40, 41].

The second dimension distinguishes between synchronous and asynchronous technologies. Synchronous digital tools involve real-time interaction between teachers and students, while asynchronous activities take place in delayed time and do not allow teachers to have simultaneous feedbacks on the learning outcomes [42]. The literature conveys that synchronous activities relate to higher social presence compared to asynchronous tools (see, for instance, [43]). On the other hand, some studies found that asynchronous teaching activities allow higher levels of reflection and metacognition by students [44, 45]. These advantages are associated with the time-related benefits of asynchronous tools. For instance, asynchronous tools allow every student to contribute to the class discussion and, in turn, foster peer learning strategies [46]. Finally, the extant literature tends to attribute positive effects to a combination of both modes of teaching in the same learning environment [45, 47].

Based on the evidence here presented, we classified digital tools into three main categories: (i) tools for synchronous teaching, (ii) tools for asynchronous teaching, and (iii) tools for communication with students and families.

### Latent Class Analysis

**Fig 1:**
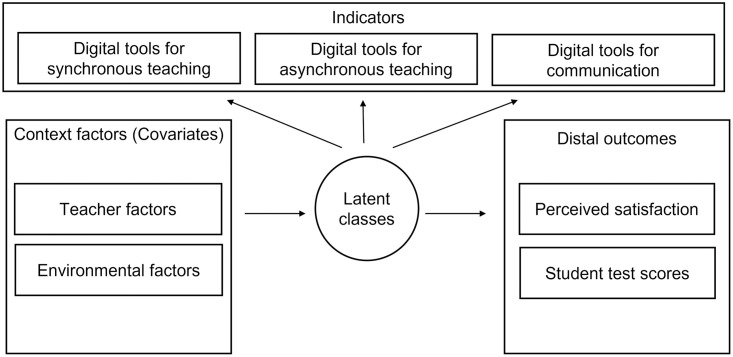
Latent Class Analysis—Measurement model.

The methodology used to explore the heterogeneity across teachers’ practices during ERT is a 3-step LCA, a mixture modelling technique increasingly used to identify latent subgroups within a population [48]. By latent subgroups, we mean clusters of observations (in this case, teachers), which share underlying common features in terms of important dimensions (in our case, the use of digital technologies). Nonetheless, the way the observations can be grouped cannot be observed directly, and so the “latent” relationship is modelled using appropriate statistical techniques. Besides the definition of latent classes (Step 1), we characterise latent classes by means of demographics and environmental factors (Step 2) and we compare them across outcomes of interest (Step 3). The LCA measurement model is summarised in Fig 1.

More specifically, in Step 1, the latent classes are defined based on 12 indicators related to the three categories defined in the digital practices’ framework (see previous section): (i) synchronous teaching, (ii) asynchronous teaching and (iii) communication with students and families. The indicators are based on questions collected through an ad-hoc survey. All the Likert-scale indicators have been dichotomised to fit the LCA algorithm, meaning that a value equal to 1 represents a frequent use of the technology (equal to a value of 4 or 5 in the original scale), while a value equal to 0 means that the technology was rarely or never used (corresponding to a value of 1, 2 or 3 in the original scale). As a result of the first step, each observation is assigned to the group for which the probability of class membership is the highest.

In Step 2, multinomial regressions are run to characterise the groups by means of personal and career-related factors. Class assignment is regressed against a set of personal and career-related covariates to provide a useful characterisation of the latent groups. In this multinomial logistic regression, one class is used as the reference group, according to which odds ratios are provided to estimate the likelihood that a certain characteristic recurs in relation to the reference group.

Step 3 makes it possible to verify whether the distal outcomes vary across groups, testing whether groups with different use of technologies report differences in teachers’ satisfaction and students’ performance (see Data Section for additional details). In this step, we compute a Pearson chi-square test to detect significant differences across groups (latent classes) obtained in Step 1 [49].

The analysis was run using Mplus version 7.4, and we followed the literature on the topic to determine the number of latent groups [50]. In this procedure, the number of classes was selected assessing the goodness of fit of the model through the Bayesian Information Criterion (BIC) and the Lo-Mendell-Rubin (LMR) test [51]. In particular, when the BIC indicates a minimum value for the K-class model, K classes should be retained. In addition, the LMR test compares the K-1 model with the K-class model. When the p-value of the LMR test is not significant anymore, then the K-1 class model should be selected. Moreover, we use entropy as an indicator of good separation between latent classes. The closer the entropy is to 1, the better the model’s specification.

## Data

### Survey design

The study is based on data gathered through a questionnaire filled in by Italian teachers during the school year 2019/20 (the ethics procedure followed the guidelines of the ethics committee of Politecnico di Milano and INVALSI; written consent was obtained from participants and data have been anonymised by INVALSI—for additional information, contact privacy@polimi.it). The survey collection started in July 2020 and was concluded at the beginning of the new school year (September 2020). Teachers were asked to answer making reference to the period of school closure lasting from February to June 2020. To guarantee comparability within grades, the questionnaires were sent out to grade 4 (primary school) and grade 7 (lower secondary school) teachers of Reading, Mathematics and English. This way, we were able to monitor the performance of the same students through the INVALSI tests in 2021, in grades 5 and 8 (in primary and secondary levels, the INVALSI scores are available only for these grades). The survey was created with the support of the National Evaluation Committee for Education (INVALSI), which selected a nationally representative sample of schools across the country. We obtained answers from 1,407 teachers, with a response rate ranging between 24% (grade 4) and 31% (grade 7) of the national sample. The moderate response rate does not affect the validity of the results since our objective is not to be representative at national level, but to identify underlying patterns in the use of digital tools during the Covid-19 emergency. Indeed, data are analysed by means of a combination of statistical descriptive methods to build a picture of the use of digital tools during the pandemic and its relationship with teachers’ satisfaction and students’ results. Nevertheless, we also analysed the differences between the group of teachers answering the survey and those who did not, to identify any potential self-selection bias. The results, reported in Supplemental information Section (see S2 Table in S1 File), do not show significant differences between the two groups in terms of observable characteristics. In particular, it is worth highlighting that students in classes where teachers responded to the survey did not perform statistically different from the ones whose teachers did not fill in the questionnaire (see S1 Fig and S3 and S4 Tables in S1 File).

### Model indicators

#### Step-1 indicators

In Table 1, we report the 12 indicators associated with the three categories of digital tools used to identify the latent classes in Step 1. The selection of the indicators (i.e., the digital tools) to be included has been carried out by performing a PCA (Principal Component Analysis), and the 12 indicators correspond to the survey items with the higher factor loadings. The PCA allows reducing data dimensionality and limiting the drawbacks associated with collinear variables. Moreover, this technique allows considering only the most relevant factors for building the latent classes (for additional details on the selection process, see also S1 Table in the S1 File).

**Table 1:** Description and basic statistics of the variables adopted for Step 1 of LCA.

Cluster of Variables	Variables’ Names	Description	Possible values	Mean *(Std Dev)*
ERT tools: Communication	Com_Wapp	How much teacher uses WhatsApp for communicating with students	From 1 (never) to 5 (always)	2.96 *(1*.*45)*
	Com_Social	How much teacher uses social media for communicating with students	From 1 (never) to 5 (always)	1.25 *(0*.*72)*
	Com_Text	How much teacher uses text messages for communicating with students	From 1 (never) to 5 (always)	1.6 *(1*.*03)*
	Com_Call	How much teacher uses phone calls for communicating with students	From 1 (never) to 5 (always)	2.69 *(1*.*14)*
ERT tools: Synchronous teaching	Syn_Slide	How much teacher uses slides in synchronous teaching	From 1 (never) to 5 (always)	2.95 *(1*.*3)*
	Syn_Video	How much teacher uses videos in synchronous teaching	From 1 (never) to 5 (always)	3.23 *(1*.*19)*
	Syn_Survey	How much teacher uses surveys/tests in synchronous teaching	From 1 (never) to 5 (always)	1.63 *(1*.*09)*
	Syn_Game	How much teacher uses learning games in synchronous teaching	From 1 (never) to 5 (always)	2.4 *(1*.*24)*
ERT tools: Asynchronous teaching	Asyn_Forum	How much teacher uses forums in asynchronous teaching	From 1 (never) to 5 (always)	2.93 *(1*.*46)*
	Asyn_Text	How much teacher uses texts (book chapters or documents) in asynchronous teaching	From 1 (never) to 5 (always)	3.99 *(0*.*94)*
	Asyn_Video	How much teacher uses videos in asynchronous teaching	From 1 (never) to 5 (always)	3.98 *(0*.*91)*
	Asyn_App	How much teacher uses learning apps in asynchronous teaching	From 1 (never) to 5 (always)	2.91 *(1*.*34)*

ERT is Emergency Remote Teaching.

#### Step-2 indicators

In the second step of the LCA, a set of control variables have been used to characterise the latent classes of teachers. In particular, we collected information on personal characteristics (e.g., age and gender), their previous training on technologies for teaching, their level of readiness for guaranteeing learning continuity (i.e., switching to online teaching soon after school closure), their relationships with colleagues, and their working environment (see details in S3 Table in S1 File). Previous literature found that the extent of the digital gap during the pandemic was linked to the gender and the age of teachers [3] and that a positive element triggered by the Covid-19 school closure was the development of new, self-organised virtual communities of teachers and school principals [6, 21, 40]. These networks enabled teachers to share good practices, thus improving their teaching experience and acquiring new skills.

#### Step-3 indicators

The survey also contains information about teachers’ satisfaction with ERT, one of the two distal outcomes of LCA. More specifically, teachers were asked to state their level of agreement with the following sentence: “In general, I am satisfied with the way I carried out teaching activities during ERT”. This variable could be partially linked to teachers’ self-efficacy of their educational activities during the emergency school closure (see, for instance, [52]). The indicator can, indeed, capture qualitative elements of students’ performance, such as motivation [53], learning autonomy [54], and school behaviour [47]. Therefore, teachers’ satisfaction can reveal a lot about the overall degree of reaction of the Italian educational system to school closure.

The second distal outcome considered in the third Step of LCA is students’ results in standardised test scores, as collected by INVALSI with reference to Reading, Mathematics and English. Teacher-level information, gathered through the survey, has been associated with the class average score in the specific subject taught by the teacher. Due to the emergency, INVALSI suspended standardised tests in 2020; therefore, we employed the students’ scores collected in May 2021. These data represent the first available information on standardised test scores of Italian students after the Covid-19 emergency. In this sense, this indicator does not capture the short-term effect of ERT on students’ performance, but it provides rather indications of a medium-term outcome.

### Descriptive statistics

The complete descriptive statistics of the variables collected through the survey and adopted in the Step 2 of LCA are reported in the S5 Table in S1 File. Of the 1,407 teachers that completed the survey, the majority are from the north of the country (52%), while 33% are from Southern Italy and the remaining 15% from Central Italy. Overall, 93% of the respondents are women, while the subjects taught are balanced across disciplines (35% English, 30% Italian and 35% mathematics). Among them, more than 90% are tenured teachers, who hold a permanent position at school.

Regarding the reaction to ERT, only less than one-third of the teachers started synchronous lessons within two weeks after school closure. Other important aspects relate to teachers’ working conditions and social environment. The majority of teachers have a quiet place of work and a personal laptop (83% of the total). They also report having had stimulating discussions with colleagues and, in most cases, they received clear guidelines from school principals on their teaching activity. On the use of digital tools, Table 1 shows that, during Covid-19, contents were delivered to students mainly through videos, for both asynchronous and synchronous activities. Almost all teachers used e-mails to communicate with students and parents, while WhatsApp was intensively used by more than 40% of teachers.

**Fig 2:**
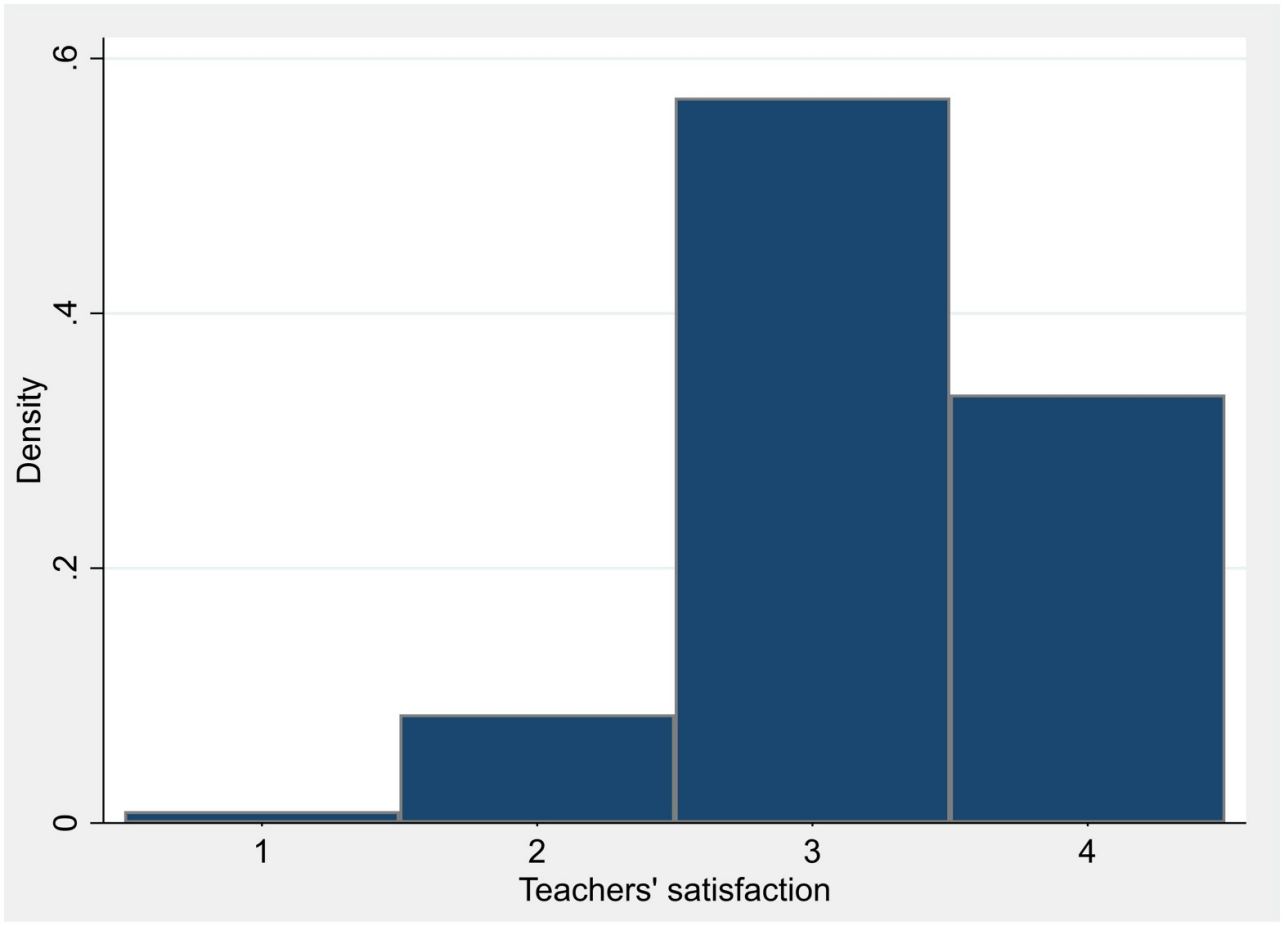
Histogram—Teachers’ satisfaction. Source: Authors’ elaboration using INVALSI 2021.

**Fig 3:**
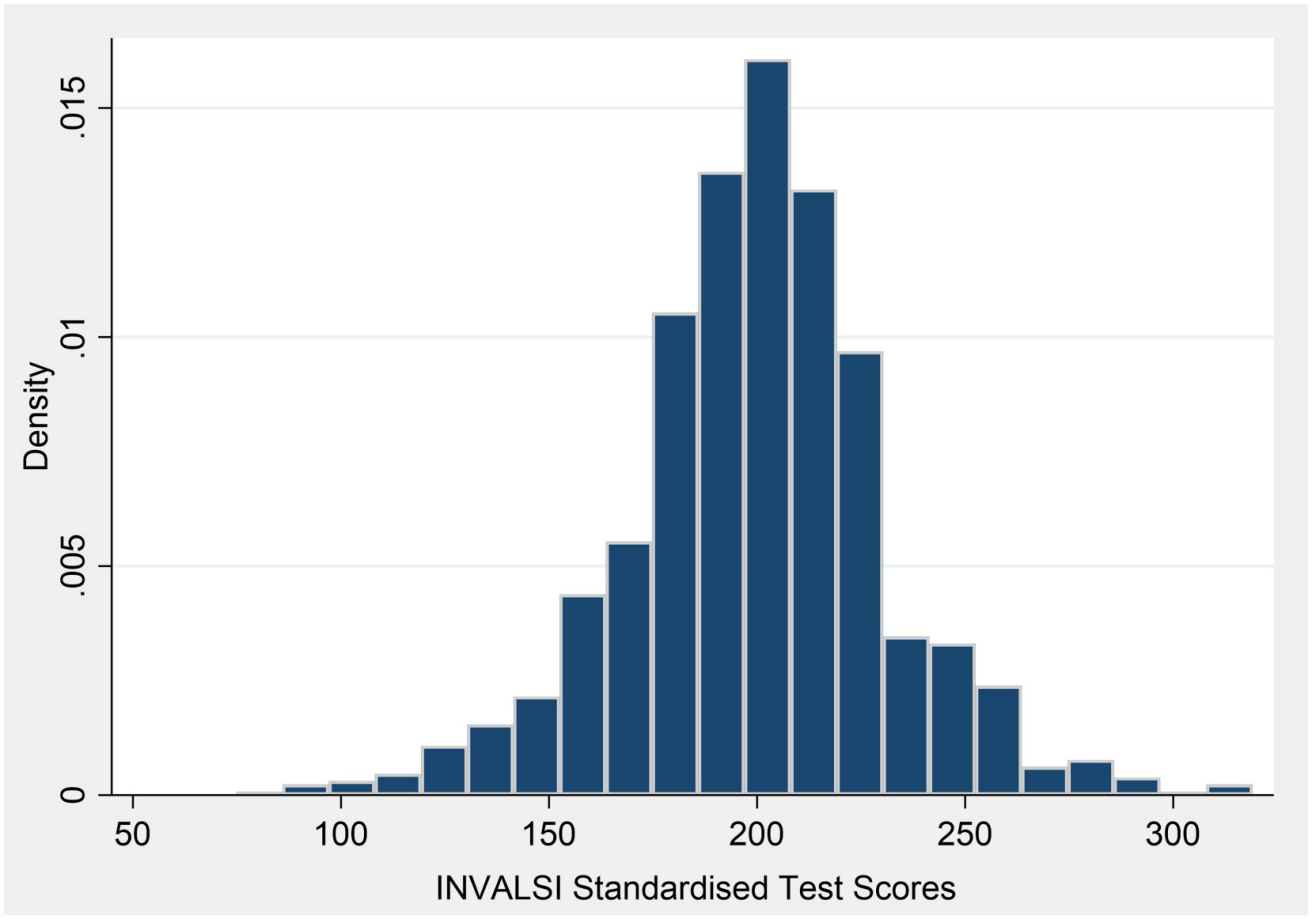
Histogram—INVALSI 2021 standardised test scores. Standardised test scores aggregated at teacher level, jointly considering Mathematics, Reding and English. Source: Authors’ elaboration using INVALSI 2021.

Concerning the outcome measures of this study, Figs 2 and 3 report the distribution of teachers’ satisfaction and students’ performance, respectively. As showed in Fig 2, teachers report a fair level of satisfaction with ERT activities (i.e., 3.23 on a 4-point Likert scale). Students’ performance, measured by aggregating the INVALSI students’ results in 2021 at teacher level, has a mean value slightly below the theoretical average (i.e., 199.7). Indeed, INVALSI standardised test scores are built to have a conventional national mean of 200 and a standard deviation of 40, following a normal distribution.

## Results

As a result of the application of the 3-step LCA to the 12 indicators related to the digital technologies used by teachers, the model fit statistics are presented in Table 2 and indicate the number of latent classes that best proxy our data. The LMR test points to a 4-class model, given that the p-value exceeds the 5% threshold in the 5-class specification. However, the BIC reaches a minimum when the number of classes is equal to 5, thus pointing to a 5-class specification model. Despite this, we opt for the more conservative and parsimonious 4-group typology model, given that the literature stresses the potential drawback of selecting too many classes [55, 56].

**Table 2:** Latent Class Analysis—Fit statistics.

Number of latent classes	AIC	BIC	LMR test	p-value	Entropy
2	16883.8	17015.0	684.54	0.00	0.570
3	16530.7	16730.1	375.15	0.00	0.648
**4**	**16399.5**	**16667.2**	**155.49**	**0.00**	**0.666**
5	16303.8	16639.8	120.40	0.06	0.668
6	18267.8	16672.0	61.41	0.15	0.716

AIC = Akaike information criterion; BIC = Bayesian information criterion; LMR = Lo-Mendell-Rubin likelihood ratio test. The p-value refers to the significance level of the LMR test.

**Fig 4:**
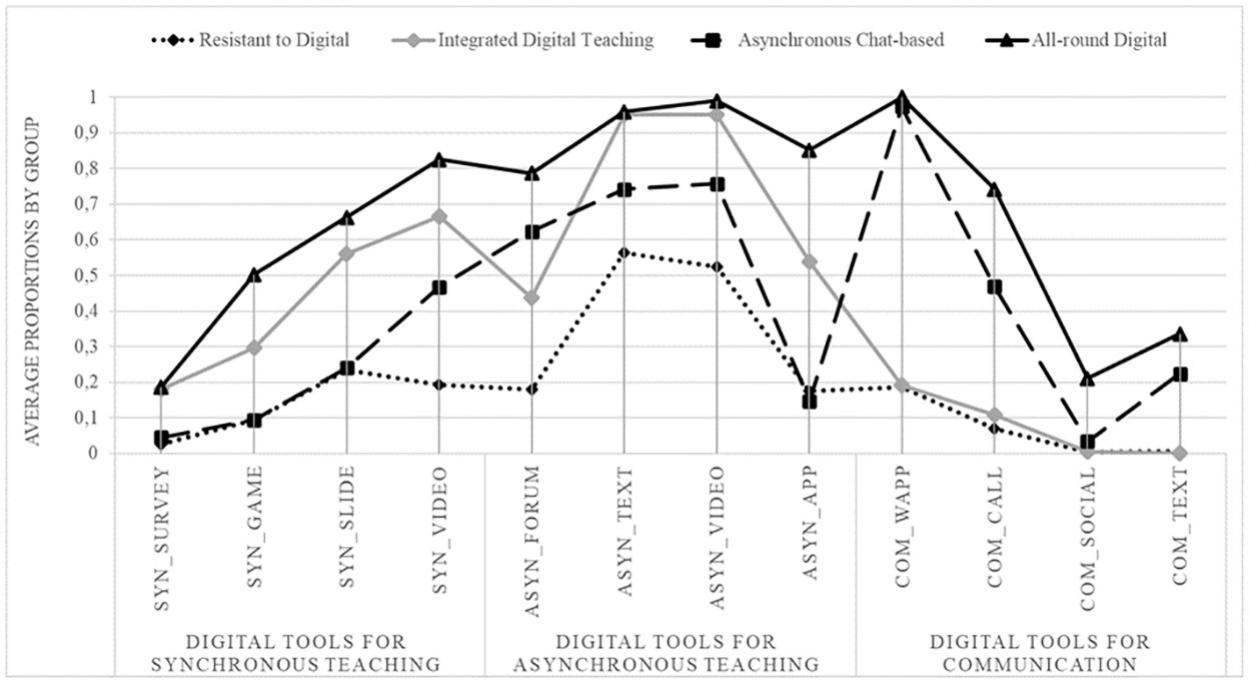
Step 1—Latent classes’ profiles. Indicators related to the use of technologies by teachers during the emergency are reported on the x axis. They are grouped into three categories related to type of adoption: for synchronous teaching, for asynchronous teaching or for communication with students and their families. Each line represents the one teachers’ profile and dots relates to the proportions of teachers belonging to that latent class. Resistant to Digital, n = 466 (33%), Integrated Digital Teaching, n = 534 (38%), Asynchronous Chat-based, n = 286 (20%), All-round Digital, n = 121 (9%).

The profile of the four classes of teachers is presented in Fig 4, where the horizontal axis details the 12 indicators on synchronous/asynchronous/communication technologies used by teachers, and the vertical axis presents the proportion of teachers who reported to have frequently used that technology during ERT. It is worth stressing that, despite having selected the technologies that explain most of the variability in the data, some of the digital tools are used to a limited extent by all the subgroups of teachers. This is particularly evident from the limited use of instant surveys in synchronous teaching, as well as from the use of social media and text messages for communication purposes. On the contrary, some digital tools have been used extensively across subgroups, and this is particularly evident for texts (e.g., book chapters and documents) and videos used for asynchronous teaching.

By analysing the profile of the four latent classes, the following subgroups emerge (to be noted: labels aim at synthetically describing their main features). The two classes at opposite poles are the *all-round digital* (9% of the observations) and the *resistant to digital* (33%) groups. The first class is made up of teachers who used a wide range of digital tools, ranging from teaching games (nearly 50% of *all-round digital* teachers adopted them) to teaching apps (nearly 80% of *all-round digital* teachers used them). Still, this group used instant surveys and quizzes, phone calls and text messages to a very limited extent.

The opposite group is made up of *resistant to digital* teachers, comprising one third of the total observations. This group used all the digital technologies to a very limited extent, thus showing some sort of resistance to digital teaching. Remarkably, only half of them shared texts or videos with students to support asynchronous teaching. All the other digital tools were used by less than 25% of the teachers. The fact that this group makes up one third of the overall number of observations is an important point to consider, as it implies that one third of the student population was stimulated very poorly by their teachers and mainly through asynchronous teaching.

The third and largest class is that of *integrated digital teaching*, which makes up 38% of the observations. This group of teachers used a wide range of digital tools and was particularly keen on using them to support the teaching activity. On the one hand, they did not use communication tools, a fact that underlines the attention specifically devoted by this group to the main task of a teacher, which is to support student learning through the teaching activity. On the other hand, this group of teachers did not pay particular attention to the communication aspects, although this was a particularly relevant matter during school closure.

The fourth and final class is *asynchronous chat-based* (20% of the observations), which has a peculiar profile related to the fact that these teachers mainly used asynchronous digital tools, either for teaching or for communicating. Indeed, to a large extent, they used asynchronous tools for teaching and WhatsApp groups to communicate with students and families. It may also be the case that these teachers used a mobile app, like WhatsApp, to assign asynchronous tasks to students, thus using a non-teaching-related digital tool for teaching activities.

The second step of the LCA aims to characterise the classes defined above through demographic, career-related and school-related information. Table 3 reports the results from the multinomial logistic regression, where the *resistant to digital* group is used as the reference group. Looking at the comparison between *integrated digital teaching* and *resistant to digital* groups, the teaching experience has a significant impact on the likelihood of resisting. Indeed, the negative coefficient states that being a more experienced teacher reduces the probability of belonging to the *integrated digital teaching* class relative to the *resistant to digital* group. In this respect, this effect may represent a propensity to use digital tools for teaching that decreases with a teacher’s experience. This finding highlights the relevance of digital competence and confidence in managing a larger use of technologies. Moreover, teachers who received training on digital technologies for teaching in the previous two years are 1.69 times more likely to conduct *integrated digital teaching* rather than being *resistant to digital*, stressing the importance of training on digital tools [58].

**Table 3:** Step 2—Characterising the latent classes.

	Integrated Digital Teaching			Asynchronous Chat Based			All-round digital			Resistant to Digital
	Coef.	Odds ratio	Mean	Coef.	Odds ratio	Mean	Coef.	Odds ratio	Mean	Mean
Age	0.01		48.18	0.004		51.14	0.028		49.76	48.24
Gender (female)	0.780*	2.18	0.93	0.712		0.94	1.977**	7.22	0.96	0.91
Central Italy	-0.496		0.13	1.024***	2.78	0.16	0.746		0.16	0.16
Southern Italy	0.202		0.26	2.236***	9.36	0.55	2.069***	7.92	0.55	0.22
Subject: Italian	-0.058		0.18	0.155		0.17	0.202		0.18	0.12
Subject: Mathematics	0.061		0.15	0.550*		0.19	-0.206		0.12	0.19
Primary school	-0.314		0.46	-0.060		0.53	0.148		0.60	0.54
Number of classes	0.012		3.84	-0.048		3.17	-0.285**	0.75	3.05	3.52
Experience (years)	-0.026*	0.97	19.42	0.018		22.19	-0.039*	0.96	20.40	20.26
Tenured teacher	0.394		0.90	-0.247		0.92	-0.910*	0.40	0.89	0.88
Managerial role	0.244		0.43	-0.327		0.31	0.467		0.45	0.36
Training on digital tools	0.527**	1.69	0.57	0.144		0.48	0.525		0.60	0.47
Digital Precovid	0.689***	1.99	3.66	-0.141		3.29	1.337***	3.81	3.88	3.29
Quite place of work	-0.015		0.82	0.425		0.88	0.485		0.92	0.81
Personal laptop	-0.169		0.84	-0.139		0.87	0.222		0.90	0.83
Discussion w/colleagues	0.154		3.37	0.145		3.28	0.288		3.42	3.29
Guidelines from SP	-0.046		3.04	-0.061		3.08	0.242		3.30	3.03
Quick start of class (after lockdown)	0.247		0.27	0.278		0.31	0.125		0.36	0.22

Coefficients result from logistic regressions.

*p≤.10.

** p≤.05.

*** p≤.01.

Odds ratios are reported for statistically significant correlation only and represent the effect of the predictors on the likelihood that one outcome will occur with respect to the reference category (Resistant to digital).

Similar findings emerge when comparing *all-round digital* to the *resistant to digital* group. In this comparison is worth stressing the relevance of supplementary personal information about the teacher. First, female teachers are 7.22 times more likely to be *all-round* rather than *resistant to digital*. However, in interpreting this result, we should keep in mind that 98% of the respondents are women (see Supporting information). Moreover, the more extensive the previous experience using digital technologies for teaching, the larger the likelihood (3.81 times) of being *all-round* rather than *resistant to digital*. Furthermore, teachers located in Southern or Central Italy are much more likely than those in Northern Italy (reference group) to be *all-round* rather than *resistant to digital*. This finding holds true for the *asynchronous chat-based* class as well, and leads us to think that teachers resistant to digital are more likely to be in northern regions. However, this finding may also suggest a larger self-selection of teachers responding to the survey in southern rather than in northern regions.

Step 3 of the LCA allows to assess whether latent classes are statistically different in terms of distal outcomes, highlighting a statistically significant difference in teachers’ satisfaction and in student test scores among latent classes. In particular, the Chi-Square tests reported in Table 4 show that the satisfaction of *resistant to digital* and *asynchronous chat-based* groups is statistically lower than that of the *integrated digital teaching* and *all-round digital* classes. Thus, the results show that teachers using digital tools to a larger extent, are also more satisfied with their teaching activities. When looking at students’ results, the differences in student test scores are significant only when comparing *integrated digital teaching* with the other groups. This class reports significantly higher student performance, meaning that the use of a wide range of digital tools is positively associated with students’ test scores. It is worth to stress that only digital tools for teaching seems to matter, given that teachers who used digital tools also for communication (the *all-round digital* teachers) do not report significantly different student performance.

**Table 4:** Step 3—Chi-Square test to investigate whether subgroups differ in their perceived satisfaction and student test scores.

	Mean of teachers’ satisfaction	Statistically different from	Mean of student test scores	Statistically different from
Resistant to Digital (1)	3.14	(2) (4)	197.35	(2)
Integrated Digital Teaching (2)	3.31	(1) (3)	206.72	(1) (3) (4)
Asynchronous Chat Based (3)	3.18	(2) (4)	193.43	(2)
All-Round Digital (4)	3.37	(1) (3)	195.66	(2)

Significance tests are Pearson chi-square. Teachers’ satisfaction is measured on 1–4 Likert scale. Student test scores is built to have an overall mean of 200 and a standard deviation of 40.

Given the relevance of the geographical factor in affecting our results (as emerged in Step 2) and given the structural differences in terms of student achievement between the high-performing Northern regions and low-performing Southern regions of Italy [57], we run an additional robustness check at Step 3. In detail, we employ an ANCOVA model in which we test jointly for the differences in students’ test scores across latent classes and across geographical regions (divided between Northern, Central and Southern Italy). Results, reported in S6 Table in S1 File, show that the *integrated digital teaching* group reports the highest student performance also when jointly controlling for the geographical regions, and that this difference is still statistically significant.

## Discussion and concluding remarks

Our findings contribute to the recently growing debate about teachers’ practices during the Covid-19 crisis [2, 5, 6, 58]. Compared with extant studies, our analysis provides empirical evidence on the role of ERT in mitigating the potential negative effects of school closure. Indeed, there is still a lack of knowledge on the role of ICT tools in mediating ERT and on the effects of ICT on learning outcomes [11]. Moreover, the paper brings attention to the crucial role that teachers play, especially during the critical period of the first wave of the Covid-19 pandemic.

Results provide new insights into how teachers addressed the online transition during the pandemic, revealing a high heterogeneity of approaches to ERT by teachers. Our findings suggest the existence of four latent classes of remote teaching behaviours during school closure. These typologies are in line with pre-Covid evidence on digital practices of teachers available in the literature [58]. More specifically, integrated digital teaching is the largest class, representing teachers that habitually used digital tools to support remote teaching activities—but not for communicating with students and families. The second class in terms of numerosity is the resistant to digital teachers, which corresponds to one-third of the sample. The high share of this typology of teachers highlights a worrisome situation, given that digital tools were the only possible channel for delivering education during the emergency. In addition, considering the effect of a potential selection bias, the share of *resistant to digital* teachers should be interpreted as a lower-bound estimate—especially in the southern and central regions, in which there is the lowest share of respondents. Asynchronous chat-based class is, instead, associated with one-quarter of the teachers, who are characterised by an intensive use of asynchronous digital tools for teaching and communication finalities. Finally, our findings reveal that less than 10% of the teachers used a wide range of digital tools in all their job activities (i.e., all-round digital teachers).

To answer the second research question, we investigate the link between the four classes of teachers’ digital behaviours and learning outcomes. The results reveal that a larger use of digital tools specifically for teaching activities is associated with both higher teachers’ satisfaction and students’ standardised test scores. This result is likely to capture the educational effects associated with teachers being mainly focused on teaching activity, rather than devoting their time to organisational and communication tasks. These teachers might have spent more time to develop learning content and have produced, therefore, a positive effect on students’ achievement. Besides, in some cases, live chat software could distract the students since creates a new communication environment with all the kids in the class [59]. On the other hand, communication remains a crucial element during an emergency. Efforts in employing digital tools for communicating with families and students seem to have generated benefits in terms of qualitative learning elements, which are captured by teachers’ satisfaction.

Overall, our findings suggest that the employment of digital tools to support remote teaching activities can play a relevant role in positively influencing student learning. From this perspective, the high presence of resistant to digital teachers represents a critical issue that should be considered in the development of teachers’ training in a post-Covid situation. Indeed, our results indicate that previous experience with digital and training on digital tools reduce the likelihood of resistance to digital technologies for teaching. This is a relevant finding to support policymakers in developing plans for teachers’ training on digital technologies and adds up to the current evidence on the relevance of teachers’ digital competence to carry out high-quality ICT-mediated teaching [14, 15].

The study has some limitations, especially in relation to the possible self-selection of teachers in the survey, affecting the external validity of results. This happened more consistently in southern regions and may affect some of the geographical-related results emerging from Step 2 of the analysis. As a potential consequence of this bias, we must interpret our finding as the optimistic picture of ERT, as we could be underestimating the number of *resistant to digital* teachers.

The results presented in the paper open the avenue for future research, which may study longitudinal data to investigate the long-term effects of ERT behaviours and monitor the evolution of teachers’ digital skills. Forthcoming studies could also enlarge the context of the analysis by investigating similar research questions from an international perspective to compare the findings across countries.

## Supporting information

S1 Fig INVALSI standardised test scores between respondents and non-respondents. (PNG)

S1 File (DOCX)
