# Supplementary figures and images for: The educational effects of emergency remote teaching practices—The case of covid-19 school closure in Italy
Source: PLoS One. 2023 Jan 25;18(1):e0280494. doi: 10.1371/journal.pone.0280494 (PMC9876279; doi:10.1371/journal.pone.0280494)

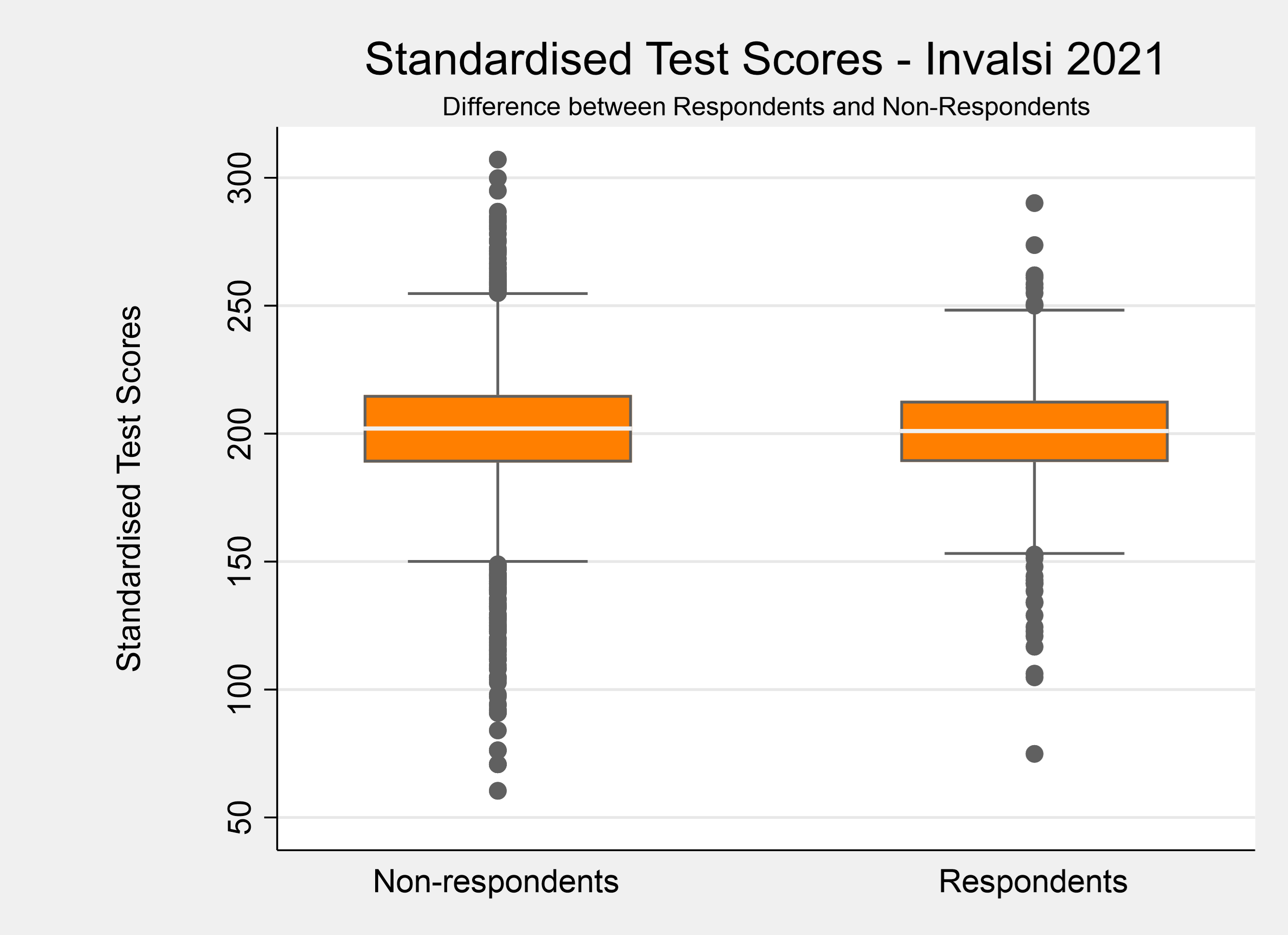

Supplement: S1 Fig — (PNG) [file pone.0280494.s001.png]
