# Supplementary material for: The educational effects of emergency remote teaching practices—The case of covid-19 school closure in Italy
Source: PLoS One. 2023 Jan 25;18(1):e0280494. doi: 10.1371/journal.pone.0280494 (PMC9876279; doi:10.1371/journal.pone.0280494)
Supplement: S1 File — (DOCX) [file pone.0280494.s002.docx]

## Results from the Principal Component Analysis

Before applying the LCA model to our data, a dimensionality reduction process is carried out by means of a Principal Component Analysis (PCA) to obtain specific indicators manageable by the algorithm. To explore the heterogeneity in teachers’ use of digital technologies, the variables involved in this step are those related to the variety of digital tools used for synchronous teaching, asynchronous teaching and for communicating with students and families. To retain the indicators to be used in Step 1 of the LCA, we focus on the first component of the PCA. Indeed, the first component is the one explaining most of the variance (specifically, more than 50%) and allows us to analyse the factor loadings. These are presented in S1 Table, in which the 12 indicators selected as inputs for the LCA are shown in bold and are those whose factor loading is greater the 0.4 (in absolute value).

**S1 Table. Loadings of the first five components from Principal Components Analysis.** Variables selected for LCA are highlighted in bold and have a factor loading greater than 0.4.

| Category | Variable | PC1 | PC2 | PC3 | PC4 | PC5 |
| --- | --- | --- | --- | --- | --- | --- |
| Synchronous teaching | **Syn_Slide** | -0.417 | 0.344 | -0.149 | 0.089 | -0.025 |
|  | Syn_Graph_Tablet | -0.255 | 0.093 | -0.144 | 0.59 | -0.277 |
|  | Syn_Excel | -0.357 | 0.127 | -0.092 | 0.611 | -0.195 |
|  | Syn_Camera | -0.28 | 0.12 | 0.002 | 0.201 | -0.103 |
|  | **Syn_Video** | -0.568 | 0.097 | -0.291 | -0.173 | 0.193 |
|  | **Syn_Survey** | -0.431 | 0.195 | -0.279 | 0.25 | 0.214 |
|  | **Syn_Game** | -0.432 | 0.105 | -0.361 | -0.132 | 0.561 |
| Asynchronous teaching | Asyn_Recording | -0.312 | -0.014 | -0.101 | 0.065 | -0.178 |
|  | Asyn_Mail | -0.266 | 0.354 | 0.716 | 0.118 | 0.137 |
|  | **Asyn_Forum** | -0.484 | -0.241 | 0.184 | -0.073 | -0.238 |
|  | **Asyn_Texts** | -0.429 | 0.275 | 0.076 | -0.39 | -0.417 |
|  | **Asyn_Video** | -0.48 | 0.193 | -0.011 | -0.487 | -0.154 |
|  | **Asyn_App** | -0.475 | 0.27 | -0.13 | -0.121 | 0.24 |
|  | Asyn_Other | 0.008 | 0.3 | 0.189 | 0.015 | 0.017 |
| Communication | Com_Mail | -0.107 | 0.498 | 0.644 | 0.067 | 0.286 |
|  | Com_Platform | -0.286 | 0.107 | -0.044 | -0.192 | -0.361 |
|  | Com_Elect_Register | -0.083 | 0.136 | 0.017 | -0.077 | -0.386 |
|  | **Com_Wapp** | -0.409 | -0.662 | 0.179 | -0.041 | 0.062 |
|  | **Com_Social** | -0.439 | -0.3 | 0.109 | 0.229 | 0.061 |
|  | **Com_Sms** | -0.419 | -0.496 | 0.221 | 0.051 | 0.067 |
|  | **Com_Call** | -0.423 | -0.53 | 0.211 | -0.056 | 0.078 |

## Testing the selection bias

The high rate of non-respondents to the survey (around 75%) could potentially lead to a selection bias. Therefore, this section aims at studying the differences between the group of teachers that responded to the survey, and one of the non-respondents. S2 Table reports the average values for the information available in the INVALSI database over the two sub-groups of teachers. The descriptive statistics show that non-respondents are equally distributed over grades and subjects. However, respondents are more likely to teach in schools situated in Northern Italy, while a higher rate of non-respondents is associated with teachers in Southern and Central of the country. Finally, statistics do not show a notable difference in terms of socioeconomic status of the class and the INVALSI Standardised Test Scores.

**S2 Table. Descriptive statistics: difference between respondents and non-respondents.**

|  | **Non Respondents** | | | **Respondents** | | |
| --- | --- | --- | --- | --- | --- | --- |
|  | Obs | Mean | Std. Dev. | Obs | Mean | Std. Dev. |
| WLE INVALSI | 3,686 | 200.9243 | 23.938 | 1,187 | 200.112 | 20.129 |
| Grade 5 | 3,761 | 58.12% | 49.34% | 1,204 | 57.64% | 49.43% |
| Reading | 3,761 | 33.71% | 47.28% | 1,204 | 32.14% | 46.72% |
| Mathematics | 3,761 | 33.08% | 47.06% | 1,204 | 34.14% | 47.44% |
| English | 3,761 | 33.21% | 47.10% | 1,204 | 33.72% | 47.30% |
| ESCS Class | 3,733 | 0.089 | 0.46 | 1,187 | 0.039 | 0.44 |
| South | 3,761 | 41.32% | 49.25% | 1,204 | 33.14% | 47.09% |
| North | 3,761 | 37.89% | 48.52% | 1,204 | 53.07% | 49.93% |
| Centre | 3,761 | 20.79% | 40.59% | 1,204 | 13.79% | 34.49% |

Further analyses have been developed for the variable on the standardised test scores since it represents one of our two outputs of interest. The box plots in S1 Fig confirm that the average scores of the classes are similarly distributed between respondents and non-respondents. The same result is statistically demonstrated by the ANOVA, which does not reject the null hypothesis of no difference in means (see S3 Table). In and S4 Table, Bartlett's test also rejects the hypothesis of non-equal variance between the two sub-groups, with a very significant p-value.

**S3 Table. ANOVA results of INVALSI standardised test scores between respondents and non-respondents.**

| **Source** | **Sum of squares** | **df** | **Mean square** | **F** | **Prob>F** |
| --- | --- | --- | --- | --- | --- |
| Between groups | 592.411689 | 1 | 592.4117 | 1.11 | 0.2914 |
| Within groups | 2592187.46 | 4871 | 532.1674 |  |  |
| Total | 2592779.87 | 4872 | 532.1798 |  |  |

**S4 Table. Bartlett's test for equal variances between respondents and non-respondents.**

| **chi2(1)** | 50.652 |
| --- | --- |
| **Prob>chi2** | 0.000 |

The analyses, here described, suggest that a potential selection bias of the respondents does not significantly influence the correlation between latent classes of teachers and students’ performance. However, we must be cautious when interpreting the results regarding non-observable characteristics, namely teachers’ digital practices and their satisfaction. Since this information is available only for the survey respondents, we cannot exclude that the respondent teachers are also the ones more incline to adopt digital behaviours and more satisfied with their remote teaching activity. In this sense, the share of *resistant to digital* teachers could have been underestimated and should be interpreted as a lower-bound estimate.

## Description of the variables

In S5 Table, we report the description of the variables employed in the second step of the LCA. These variables represent the controls used to describe the latent classes, which have been identified in the first step. The descriptive statistics are commented in the main body of the paper (see Section 3.2).

**S5 Table. List of variables adopted for 2^nd^ step of LCA, their description and basic statistics.**

| Cluster of Variables | Variables' Names | Description | Possible values | Mean  *(Std Dev)* |
| --- | --- | --- | --- | --- |
| Dependent var. | Satisfaction | General satisfaction about digital teaching | From 1 (no agree), 4 (strongly agree) | 3.23 *(0.64*) |
| Demographic information | Age | Teacher's age | Numerical value | 48.94 *(0.64)* |
|  | Gender | Teacher's gender | 1 if female, 0 otherwise | 0.93 |
|  | Northern Italy | Teacher's school region | 1 if North, 0 otherwise | 0.52 |
|  | Central Italy | Teacher's school region | 1 if Centre, 0 otherwise | 0.15 |
|  | Southern Italy | Teacher's school region | 1 if South, 0 otherwise | 0.33 |
|  | Subject: English | Subject of teaching | 1 if English, 0 otherwise | 0.35 |
|  | Subject: Italian | Subject of teaching | 1 if Italian, 0 otherwise | 0.30 |
|  | Subject: Mathematics | Subject of teaching | 1 if Mathematics, 0 otherwise | 0.35 |
|  | Primary School | School level | 1 if Primary, 0 Middle school | 0.51 |
| Career information | Number Of Classes | Teacher's number of classes | Numerical value | 3.53 *(3.51)* |
|  | Experience (Years) | Teacher's number of years of teaching | Numerical value | 20.35 *(10.22)* |
|  | Tenured Teacher | If the teacher is tenured (1, 0 otherwise) | 1 if yes, 0 otherwise | 0.90 |
|  | Managerial Role | If the teacher has a managerial role within the school (1, 0 otherwise) | 1 if yes, 0 otherwise | 0.38 |
| Digital Technologies background | Training on digital tools | If the teacher follows training courses for digital teaching | 1 (never), 2 (more than 2 years ago) or 3 (within the last 2 years) | 2.31 *(0.81)* |
|  | Digital Precovid | How much the teacher uses digital tools before COVID-19 emergency | From 1 (never) to 5 (always) | 3.48 *(0.86)* |
| Working environment during emergency | Quick start of class (after lockdown) | If the teacher starts synchronous classes within 2 weeks of lockdown | 1 if yes, 0 otherwise | 0.27 |
|  | Quite place of work | Teacher's quite working space at home | 1 yes, 0 otherwise | 0.83 |
|  | Personal laptop | If teacher has a personal device for working | 1 yes, 0 otherwise | 0.85 |
|  | Discussion w/colleagues | Teacher's discussion with colleagues about distance learning | From 1 (no agree), to 4 (strongly agree) | 3.33 *(0.69)* |
|  | Guidelines from SP | Teacher receives clear indication from School Principal during the emergence | From 1 (no agree), to 4 (strongly agree) | 3.07 *(0.86)* |

## Robustness check for Step 3

As a robustness check for Step 3, we report the results from an ANCOVA model in which we test whether students’ performance differ across latent subgroups of teachers, by jointly controlling for the geographical macro-area (i.e., Northern, Central or Southern Italy). The sample refers to the 1407 teachers that compose our reference group. Results report an F-statistic of 9.91, with a p-value of 0.000, showing that there is a statistical difference in student achievement. When running the related regression, as reported in S6 Table, we can observe that the geographical component matters, and in particular teachers in Southern regions report lower student achievement. However, the latent class variable still matters as well, and in particular the *Integrated digital teaching* class significantly outperform in terms of student achievement (especially compared to the *resisting to digital* class, used as reference).

**S6 Table. Regression run based on the ANCOVA model.**

| Output: Student test score | Coef. | Std. Err. | t | P>t | [95% Conf. Interval] | |
| --- | --- | --- | --- | --- | --- | --- |
| Class |  |  |  |  |  |  |
| Integrated digital teaching | 6.633 | 2.164 | 3.060 | 0.002 | 2.386 | 10.880 |
| Asynchronous chat based | -0.130 | 2.621 | -0.050 | 0.960 | -5.274 | 5.013 |
| All-round digital | 1.457 | 3.512 | 0.410 | 0.678 | -5.435 | 8.348 |
| Geographical macro-area | | |  |  |  |  |
| Central Italy | 0.564 | 2.740 | 0.210 | 0.837 | -4.812 | 5.939 |
| Southern Italy | -10.158 | 2.113 | -4.810 | 0.000 | -14.304 | -6.012 |
| Constant | 200.441 | 1.715 | 116.900 | 0.000 | 197.077 | 203.805 |
